# Delineation of genotype × environment interaction for identification of stable genotypes for tillering phase drought stress tolerance in sugarcane

**DOI:** 10.1038/s41598-021-98002-y

**Published:** 2021-09-20

**Authors:** C. Mahadevaiah, Prakash Hapase, V. Sreenivasa, Ramesh Hapase, H. K. Mahadeva Swamy, C. Anilkumar, K. Mohanraj, G. Hemaprabha, Bakshi Ram

**Affiliations:** 1 Division of Crop Improvement, ICAR-Sugarcane Breeding Institute, Coimbatore, India; 2 Vasantdada Sugar Institute, Pune, India; 3 ICAR-National Rice Research Institute, Cuttack, India

**Keywords:** Plant breeding, Plant breeding

## Abstract

Sugarcane is a trans-seasonal long-duration crop and tillering phase (60–150 days) is the most sensitive phase for moisture stress, causing significant reduction in biomass accumulation. The study focussed to assess the Genotype × Environment Interaction (GEI) for tillering phase moisture stress and to identify the stable genotypes in sugarcane. The study dealt with 14 drought tolerant genotypes and two standards (Co 86032 and CoM 0265) which were evaluated in two plant and one ratoon trials at four locations in Maharashtra, India. The moisture stress was imposed for 60 days from 90 to 150 days after planting and corresponded to tillering phase by withholding the irrigation. The AMMI ANOVA showed significant GEI for cane and CCS yield accounting 18.33 and 19.45 percent of variability respectively. Drought and genotype main effects were highly significant accounting 49.08 and 32.59 percent variability for cane yield and, 52.45 and 28.10 percent variability for CCS yield respectively. The first two interactive principal component (IPCA) biplots of AMMI showed diverse nature of all four environments and the Discriminative *vs* Mean biplots of Genotype + genotype × environment interaction (GGE) model showed that ‘Pune’ as the highly discriminating environment. The genotype ranking biplots of GGE showed that Co 85019 was the most stable genotype followed by Co 98017. Similar results were also observed in Yield *vs* IPCA1 biplot of AMMI, which revealed Co 85019 and Co 98017 as high yielding stable varieties. Yield related environmental maximum (YREM) showed thirteen and nine percent loss due to crossover interactions in Co 85019 for cane yield and CCS yield respectively. The multi-environment BLUP and genotype stability index (GSI) has reaffirmed that Co 85019 as a drought proof and stable genotype with high yield under tillering phase drought stress. The results suggested using Co 85019 for cultivation in drought prone regions and the usefulness of the methodology for identifying more such sugarcane varieties for the benefit of resource poor famers in drought affected regions.

## Introduction

Sugarcane is a major agro-industry based crop serving as raw material for the production of sugar and has high potentiality for renewable energy production such as biofuel and cogeneration. The moisture stress or drought is a major constraint in rainfed sugarcane cultivation due to variation and quantum of rainfall caused by global climate change and *El-Nino* southern oscillations^1–3^. Moisture stress or drought is a recurrent phenomenon with high frequency in the world^3,4^. The rainfall distribution pattern shows variation and spring/summer season goes as dry spells in most of the sugarcane growing countries^5–8^. Therefore, rain dependent sugarcane crop generally suffers due to moisture stress during spring/summer seasons. The development of drought tolerant sugarcane varieties enhances the productivity and sustainability of rainfed sugarcane agriculture.

The tillering phase is the most sensitive stage for moisture stress in sugarcane^9–14^, causing significant reduction in cane yield upto 49% through the reduction in its attributing traits such as shortening of internodes (45.76%), reduction in stalk length (45.76%), cane weight (25.5%) and the number of millable cane (22.8%)^15,16^. Development of climate resilient varieties are essentially required for effective mitigation of drought stress and for sustainable sugarcane agriculture in drought prone area^17^. The multi-environment evaluation of drought tolerant genotypes helps to understand the GEI and help to select the drought tolerant stable genotypes^16^.

The AMMI and GGE biplots models are popularly used to understand the pattern of GEI and to assess the stability of genotypes^18–26^. AMMI, a multiplicative model estimates the GEI components based on multidimensional approaches and is highly discriminative to estimate GEI components^23,25,26^. GGE Biplot analysis helps in the identification of mega environments, stable ranking of genotypes, and identification of ideal stable varieties^27,28^. The multi-locational evaluation of genotypes under tillering phase drought stress helps to assess the pattern of GEI and to identify the stable genotypes. The sugarcane is a water loving crop^29^ and cane yield under drought is one of the accurate indicators of drought tolerance^30^.

ICAR-Sugarcane Breeding Institute, Coimbatore is a premier research institute actively involved in the development of sugarcane varieties for high biomass, superior juice quality, and biotic and abiotic stress tolerance suitable for various agro climatic zones^31,32^. In order to identify the drought tolerant clones based on cane yield, fourteen drought tolerant clones and two standards (Co 86032 and CoM 0265) were evaluated under tillering phase drought stress in four locations of sugar factories research farms in collaboration with Vasantdada Sugar Institute, Pune. This programme was formulated based on recommendations of i) an Interface meeting between the Indian Council of Agriculture Research (ICAR), Ministry Agriculture and Cooperation, Government of India, and Vasantdada Sugar Institute, Pune to evaluate the drought tolerant genotypes identified^10,15,33^ at ICAR-Sugarcane Breeding Institute, Coimbatore, ii) Indian Parliamentary question on the impact of drought on sugarcane crop damage and farmers and, iii) VIII ICAR Regional Committee Meeting on development of drought tolerant and water efficient varieties. Our studies showed the significant GEI for cane and CCS yield and identified Co 85019 as the high yielding and stable variety under tillering phase drought stress for its cultivation in the water stressed regions of Maharashtra.

## Material and methods

### Material and experimental design

Fourteen drought tolerant sugarcane clones were identified at ICAR-Sugarcane Breeding Institute, Coimbatore, Co 86032 (notified variety for cultivation in Peninsular India) and CoM 0265 (notified variety for cultivation in Maharashtra)^34^ constituted the experimental material for the study. The fourteen testing clones namely Co 0238, Co 05001, Co 05007, Co 08020, Co 10017, Co 10024, Co 13003, Co 2000-10, Co 85019, Co 90003, Co 92020, Co 93009, Co 94005, Co 98017 were commercial type sugarcane genotypes developed by selection in the biparental and general cross populations (Supplementary table 1). These clones were selected based on screening for various abiotic stress tolerances at ICAR-Sugarcane Breeding Institute, Coimbatore, and selected based on the criteria of improvement in comparison with standard varieties. Co 85019 was selected for traits such as high cane yield under formative phase drought stress^15,33^, high water use efficiency and water productivity^35^ and high physiological efficiency related traits such as canopy temperature deficient, chlorophyll efficiency, leaf rolling index^36^ under water limited conditions. Co 85019 was also identified as a salinity tolerant clone, recorded high biomass accumulation at formative phase stress, and produced the high cane yield under salinity stress^37–39^ and further also used for characterization of saline responsive genes^40^. Co 98017, Co 2000-10 and Co 90003 were selected based on their superior performance for cane yield under formative phase drought stress in Coimbatore^15,33^. Co 0238, a notified variety for North-West Zone in India, cultivated in 2.5 million hectares in sub-tropical regions (80 percent of sugarcane cultivated area in sub-tropical India) and benefiting the millions of farmers in India. Further details of clones used in the study are given in supplementary table 1.

The experiments were planted in Randomized Block Design with two replications at four locations in research farms of sugar factories (Supplementary table 2). Each clone was planted in six rows of six meter length with row-to-row spacing of 1.20 m. The recommended seed rate of 12 buds per meter was planted at all locations. All these four experimental locations are located in Deccan plateau regions of India having black cotton soil or Vertisol^41^. The chemical properties of soil are given in the supplementary table 3. The recommended doses of nitrogen (340 kg/ha), phosphorous (170 kg/ha), and potassium (170 kg/ha) were adopted in all four locations. The complete dose of phosphorous was applied as basal at the time of planting, nitrogen and potassium were applied in two split doses on 45 days at the time of partial earthing-up and 90 days at the time of complete earthing-up operations^42^. All the recommended cultivation practices were adopted and trials were maintained by the respective sugar factory officials in participatory mode and, monitored by the researchers of ICAR-Sugarcane Breeding Institute, Coimbatore, and Vasantdada Sugar Institute, Pune in various stages of crop growth. The experiments were conducted in compliance with relevant institutional, national, and international guidelines and legislations.

### Imposition of moisture stress during tillering phase and weather data

The drought was imposed during tillering phase by withdrawing the irrigation from 90 to 150 days after planting/ratooning at all locations during 2018 and 2019. The weather data for all four locations was obtained from India Meteorological Department, Pune (https://www.imdpune.gov.in). The weather parameters during the drought imposition period, grand growth period, and maturity phase were given in supplementary table 4. The nil or minimum rainfall of 1.21, 4.18, 4.79, 23.48 mm during 2018 and 6.26, 3.69, 2.45, 3.95 mm of rainfall during 2019 during the drought imposition period (April 01 to May 30 of both the years) combined with high temperature and low humidity was recorded at Kopargaon, Jalna, Pune, and Nanded respectively. The low or no rainfall during this period is clearly indicated the severity of drought and justifies the need of experimentation and development of drought tolerant varieties for these regions.

### Recording of observations

Cane yield (t/ha) was estimated based on cane yield from the middle four rows of each plot. NMC was counted from middle four rows at the time of harvest. Randomly selected five canes were averaged for recording the SCW, SL and ST. The stalk length was measured from ground level to the first node of visible dewlab (Supplementary table 5). For juice analysis, five canes were randomly sampled from the clumps at 10th and 12th months and, used for recording the brix and sucrose content by using Brix Hydrometer and Polarimeter respectively. The CCS (%) and CCS yield (t/ha) were estimated as given below^43–45^.

$$\begin{aligned} Brix \left( \% \right) & = \left( {\left( {0.77 \times (temperature\; in\;{^\circ }C-27.5)} \right) + Observed \,brix} \right) \\ Sucrose \left( \% \right) & = \left( {\left( {pol\, value \times 0.26} \right) - \left( {\frac{pol\, value}{{1000}} \times \left( {brix - 1} \right)} \right)} \right) - 0.02 \\ Purity \left( \% \right) & = \frac{Sucrose \left( \% \right)}{{Brix \left( \% \right)}} \times 100 \\ CCS\left( \% \right) & = \left( {Sucrose\% \times {1}.0{22}} \right) - \left( {Brix\% \times 0.{292}} \right) \\ CCS\, Yield \left( {t/ha} \right) & = \frac{{CCS\left( \% \right) \times Cane\, yield \left( {t/ha} \right)}}{100} \\ \end{aligned}$$

### Statistical analysis

The replication-wise data of plant and ratoon trials from each location were subjected to statistical analysis. The homogeneity of error mean variance was done by Levene’s test^46^ for all 10 experiments using SPSS 16 (IBM Corp. Released 2016). The non-significance of Levene’s statistics for all traits revealed the homogeneity of error mean variance across all locations for further statistical analysis.

#### BLUP estimation

The BLUP for each location and multi environment BLUP were estimated in restricted maximum likelihood method using mixed linear model of lme4 package of meta-R^47^. The mixed linear model for Randomised Block Design with replications for each location is defined as

$$\mathrm{Yij}=\upmu +\mathrm{Ri}+\mathrm{Gj}+{\sf C}\!\!\!\!\raise.8pt\hbox{=}{ij}$$

where Y_ij_ is response variables like cane yield and CCS yield, µ is overall mean effect, R_i_ is ith replicate effects, G_j_ is jth genotype effect and €ij the effect of variance associated with ith replicate and jth genotype.

The mixed linear model for multi-environment BLUP for Randomised Block Design^47^ with replications by the inclusion of new factor ‘Drought Environments is as follows

$$\mathrm{Yijk}=\upmu +\mathrm{Lk}+\mathrm{RiLk}+\mathrm{Gj}+\mathrm{Lk}\times \mathrm{Gj}+{\sf C}\!\!\!\!\raise.8pt\hbox{=}{ijk}$$

where Y_ijk_ is response variables like cane yield and CCS yield, µ is average effect of response variable, L_k_ is kth location effect, R_i_ is ith replication effect at kth location, G_j_ is jth genotype effect, €_ijk_ is the unknown effect related to ith replication jth genotype kth drought environments.

The heritability (broad-sense) for a given trait^47^ for each drought location was estimated as

$${\mathrm{H}}^{2}=\frac{{\upsigma }_{\mathrm{g}}^{2}}{{(\upsigma }_{\mathrm{g}}^{2}+\frac{{\upsigma }_{{\sf C}\!\!\!\!\raise.8pt\hbox{=}}^{2}}{\mathrm{nR}})}$$

where H^2^ is broad-sense heritability for a given trait, σ^2^_g_ is genotypic variance for trait of interest, σ^2^_€_ is residual variance and nR is replication numbers.

The broad-sense heritability for a given traits for the combined drought environments^47^ was estimated as

$${\mathrm{H}}^{2}=\frac{{\upsigma }_{\mathrm{g}}^{2}}{{(\upsigma }_{\mathrm{g}}^{2}+\frac{{\upsigma }_{\mathrm{ge}}^{2}}{\mathrm{nL}}+\frac{{\upsigma }_{{\sf C}\!\!\!\!\raise.8pt\hbox{=}}^{2}}{\mathrm{nL}\times \mathrm{nR}})}$$

where H^2^ is broad-sense heritability for a given trait, σ^2^_g_ is genotypic variance for trait of interest, σ^2^_ge_ is genotypic variance due to GEI for trait of interest, nL is number of drought environments and nR is replication numbers.

The genetic correlations (ρg_ij_) between traits ‘i' and ‘j’ across drought environments^47^ by using the variance component was estimated as

$${\uprho }_{{\mathrm{g}}_{\mathrm{ij}}}=\frac{{\upsigma }_{\mathrm{ij}}^{2}}{{\upsigma }_{\mathrm{gi}}+{\upsigma }_{\mathrm{gj}}}$$

where

$$\sigma_{gi} = \sqrt {\sigma_{gi}^{2} } \;{\text{and}}\;\sigma_{gj} = \sqrt {\sigma_{gj}^{2} }$$

where σ^2^_gi_, σ^2^_gj_ and σ^2^_ij_ are ith and jth traits genetic variances and genetic covariances respectively.

The BLUP was calculated by meta-R^47^ by treating the genotypes as random effect and drought location as fixed effect.

#### AMMI and GGE Biplot analysis

AMMI stability model calculates drought environment and genotype main effect and, multiplicative effects of GEI. The AMMI stability analysis was done using GEA-R^48^ and the model^49^ aswhere the response variable such as cane yield and CCS yield is represented by Y_ijk_, grand mean represented by µ, genotype deviation from µ represented by G_i_, environment deviation from µ represented by E_j_, eigen value of kth interactive principal component (IPCA) represented by ʎ_k_, IPCA score for ith genotype on kth IPCA represented by α_ik_, IPCA score of jth environment for kth IPCA represented by ɤ_jk_, residual GEI unexplained by model represented by d_ij_ and model error represented by e_ijk_. The sum of square (SS) due to GEI due to signal^22^ was estimated as

$${\mathrm{SS}}_{\mathrm{Signal}}={\mathrm{SS}}_{(\mathrm{GEI})}-{\mathrm{SS }}_{\mathrm{Noise}}$$

$${\mathrm{SS}}_{\mathrm{Noise}}={\mathrm{degree\, of \,freedom}}_{\left(\mathrm{GEI}\right)}\times \mathrm{Mean \,squares\, of \,residuals}$$

The GGE Biplots such as ranking of environments and genotypes, mean vs stability biplots based on average performance of genotype for cane yield and CCS yield with stability, discriminativeness vs representativeness biplots based on evaluation of environments with their discriminative ability and which own where biplots for identifying the cultivars for given environments by using GEA-R package.

The stable performance of genotypes in AMMI stability model was calculated as AMMI Stability values (ASV)^18^ as

$$ASV=\sqrt{{\left[\frac{{SS}_{IPCA1}}{{SS}_{IPCA2}} \times \left({IPCA}_{1}\right)\right]}^{2}+{\left({IPCA}_{2}\right)}^{2}}$$

where SS_IPCA1_ is sum of square due to first interaction component, SS_IPCA2_ is sum of square due to second interaction component and, IPCA_1_ and IPCA_2_ are sum of score for first and second interaction component for each genotype. The genotype stability index (GSI) was used to compare stability of high cane and CCS yielding genotypes and ranking the genotypes by combining both yield and stability parameters^21^. The GSI for each genotype was estimated as

$${GSI}_{i}={rY}_{i}+{rASV}_{i}$$

where GSI is genotype selection index for ith genotype^21^, rY_i_ is derived from rank of adjusted mean for cane and CCS yield for genotype ‘i' and rASVi is derived based on ASV rank of genotype ‘i'. The yield related environmental maximum (YREM) was used to compare the genotypes for yield loss due to the crossover interaction of genotype and environments^50,51^. The cane yield and CCS yield related to each environmental maximum was calculated as follows 

$${Y}_{ij}=\frac{{X}_{ij}}{{MAX}_{j}}$$

where Y_ij_ is cane yield and CCS yield related environmental maximum for genotype ‘i' in drought environment ‘j’, X_ij_ is the average cane and CCS yield of genotype ‘i' in drought environment ‘j’ and MAX_j_ is drought environmental maximum recorded for cane yield and CCS yield in environment ‘j’.

## Results

### Best linear unbiased prediction combined all locations for cane yield and CCS yield

The BLUP is the most accurate estimate of the random genetic effects in linear mixed models and has gained popularity in varietal testing in plant breeding^52,53^. The BLUP allows unbiased estimates of random genetic effects and enhances the reliability of estimated breeding value and heritability^53^. Comparing genotypes performance over years (time) and environments (space) with simultaneous correction of environmental effects and prediction of real genotypic effects is an advantage of BLUP approach^54^. In our studies, the estimates of multi-environment BLUP was highly significant for all traits. Heritability estimates ranged from 0.513 for sucrose content to 0.748 for cane yield, showing relatively high heritability for all traits (Table 1). The estimates of genetic correlation showed that both cane yield, and CCS yield had significant positive correlation with SL (0.969, 0.939), SCW (0.875, 0.796) and NMC (0.817, 0.868) respectively (Table 2). The estimates of genotypic and drought variances were greater than variance due to GEI for all the traits indicating that significant role of genotype, tillering phase drought, and GEI contributing to the trait expression.

**Table 1:** Estimates of genetic parameters from multi environment BLUPs for cane yield, CCS yield and components under tillering phase drought stress at ten test drought environments.

Statistic	BLUP_CY	BLUP_CCSY	BLUP_Suc	BLUP_NMC	BLUP_SL	BLUP_ST	BLUP_SCW
Heritability	0.748	0.709	0.513	0.588	0.604	0.746	0.722
Genotype variance	172.431	2.784	0.202	33.710	160.864	0.023	0.024
GEI variance	111.593	2.351	0.118	19.491	97.940	0.007	0.009
Residual variance	241.881	4.784	0.917	102.563	646.314	0.049	0.056
Grand mean	80.488	10.834	18.373	62.258	203.623	2.824	1.320
LSD	16.286	2.387	0.794	8.851	18.646	0.169	0.188
CV	19.323	19.454	5.212	16.267	12.485	7.862	17.850
Genotype significance (p value)	0.000	0.000	0.024	0.003	0.001	0.000	0.000
G x E significance (p value)	0.000	0.000	0.110	0.011	0.007	0.023	0.005

*BLUP* best linear unbiased prediction, *CY* cane yield (t/ha), *CCSY* commercial cane sugar yield (t/ha), *Suc* sucrose percent at 12th month, *SL* stalk length (cm), *ST* stalk thickness (cm), *SCW* single cane weight (kg), *NMC* number of millable canes, *LSD* least square difference, *CV* coefficient of variation.

**Table 2:** Estimates of genotypic correlation between cane yield, CCS yield and component traits across ten test drought environments under drought stress in sugarcane.

Traits	CY	CCSY	Suc	SL	ST	SCW
CCSY	0.987**					
Suc	− 0.315	− 0.141				
SL	0.969**	0.939**	− 0.390			
ST	0.420	0.315	− 0.674**	0.305		
SCW	0.875**	0.796**	− 0.632**	0.872**	0.771**	
NMC	0.817**	0.868**	− 0.246	0.540*	− 0.026	0.323

**significant at 1% level; *significant at 5% level.

*CY* cane yield (t/ha), *CCSY* commercial cane sugar yield (t/ha), *Suc* sucrose percent at 12th month, *SL* stalk length (cm), *ST* stalk thickness (cm), *SCW* single cane weight (kg), *NMC* number of millable canes.

**Figure 1:**
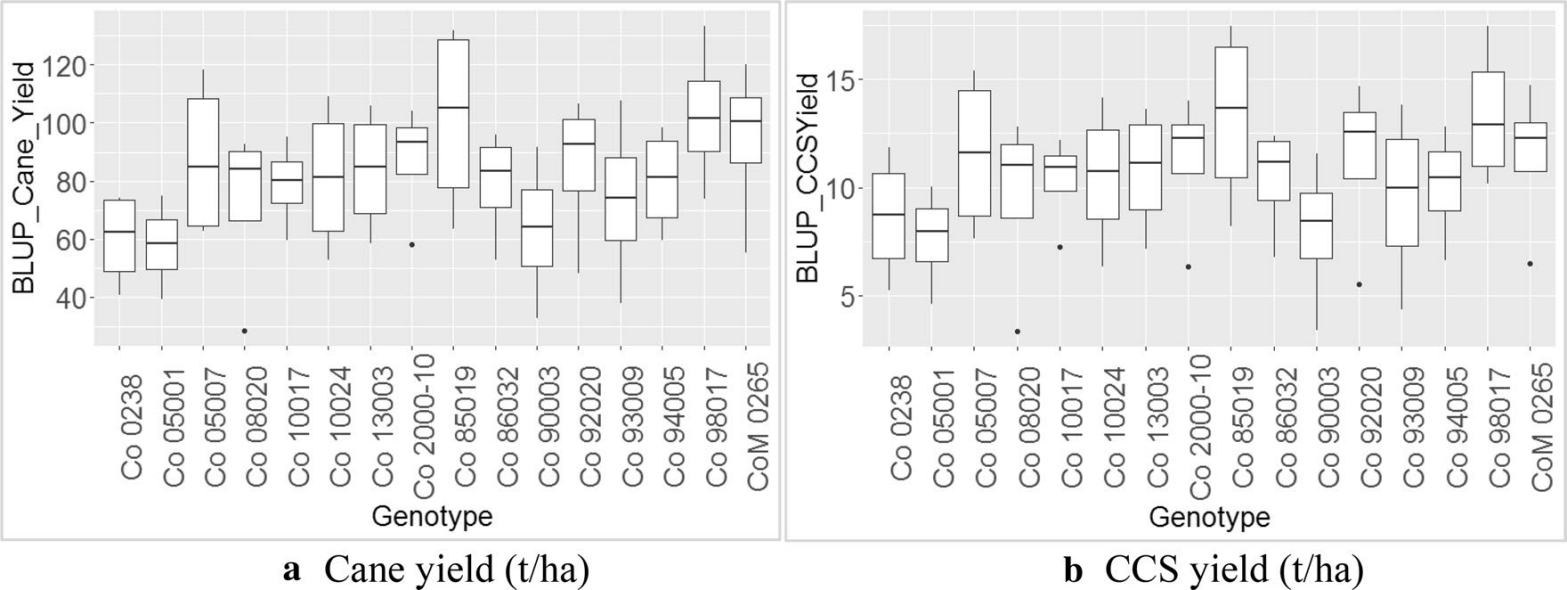
Box plot depicting the multi-environment BLUP based performance for cane yield and CCS yield under tillering phase drought stress of sugarcane genotypes across locations**. (a**) Multi-environment BLUP depicting the superior performance of Co 85019 for cane yield under tillering phase drought stress, (**b**) Multi-environment BLUP depicting the superior performance of Co 85019 for CCS yield (t/ha) under tillering phase drought stress.

Treating the BLUP estimated genotype as random effect and location as fixed effect revealed the highest multi-environment BLUP for cane yield (Table 3 and Fig. 1a) recorded by Co 85019 (99.41 t/ha) followed by Co 98017 (99.31 t/ha) and standard variety CoM 0265 (93.37 t/ha). Similarly, multi-environment BLUP has shown that Co 85019 (12.93 t/ha) was the best clone for CCS yield (Table 4 and Fig. 1b) followed by Co 98017 (12.92 t/ha), Co 05007 (11.39 t/ha) and the standard variety CoM 0265 (11.37 t/ha). The BLUP is reported to outperform in the selection of stable varieties^52^ and in our studies; Co 85019, Co 98017 and Co 05007 were identified as promising drought tolerant sugarcane clones.

**Table 3:** Comparative mean performance, BLUP, AMMI stability indices for cane yield (t/ha) under drought stress in sugarcane.

Genotypes	Mean	BLUP-CY	AMMI adjusted mean	ASI	rASI	rCY	GSI	YREM
Co 0238	57.93	61.72	57.42	0.3260	2	15	17	0.49
Co 05001	54.11	59.88	55.27	0.1580	1	16	17	0.47
Co 05007	87.75	86.59	88.50	1.0719	13	4	17	0.73
Co 08020	71.00	73.88	71.80	1.5289	15	13	28	0.63
Co 10017	79.90	78.94	78.35	0.5543	8	11	19	0.68
Co 10024	81.12	80.86	81.22	0.5953	9	8	17	0.67
Co 13003	84.06	83.07	83.86	0.3581	3	7	10	0.70
Co 2000-10	89.95	86.91	87.98	0.7750	11	5	16	0.77
Co 85019	104.26	99.41	103.74	0.4395	6	1	7	0.87
Co 86032	82.61	79.26	78.71	0.4459	7	10	17	0.68
Co 90003	66.71	64.92	61.49	0.3691	4	14	18	0.52
Co 92020	85.33	85.05	85.62	0.7428	10	6	16	0.74
Co 93009	76.17	74.39	73.19	1.1112	14	12	26	0.62
Co 94005	76.94	80.04	79.93	0.4120	5	9	14	0.68
Co 98017	101.06	99.31	102.05	1.8370	16	2	18	0.86
CoM 0265	98.73	93.37	95.69	0.9810	12	3	15	0.83

*BLUP* best linear unbiased prediction, *CY* cane yield (t/ha), *ASI* AMMI stability index, *rASI* rank of AMMI stability index, *rCY* rank of cane yield, *GSI* genotype selection index, *YREM* yield related environmental maximum.

**Table 4:** Comparative mean performance, BLUP, AMMI stability indices for CCS yield (t/ha) under drought stress in sugarcane.

Genotypes	Mean	BLUP-CCSY	AMMI adjusted mean	ASV	rASV	rTM	rGSI	YREM
Co 0238	8.38	8.82	8.37	0.594	8	13	21	0.54
Co 05001	7.26	7.93	7.19	0.135	1	15	16	0.48
Co 05007	11.70	11.39	11.73	1.051	13	4	17	0.77
Co 08020	9.92	9.78	9.48	1.584	16	11	27	0.65
Co 10017	10.49	10.32	10.24	0.448	6	9	15	0.70
Co 10024	10.54	10.47	10.52	0.548	7	7	14	0.68
Co 13003	10.85	10.69	10.79	0.433	5	6	11	0.70
Co 2000-10	11.70	11.17	11.31	0.727	10	5	15	0.78
Co 85019	13.85	12.93	13.66	0.715	9	1	10	0.91
Co 86032	10.53	10.37	10.32	0.215	2	8	10	0.69
Co 90003	7.75	8.29	7.66	0.267	3	14	17	0.51
Co 92020	11.89	11.3	11.45	0.739	11	3	14	0.77
Co 93009	9.60	9.72	9.52	1.219	14	12	26	0.63
Co 94005	10.21	10.1	9.96	0.404	4	10	14	0.68
Co 98017	13.54	12.92	13.80	1.539	15	2	17	0.89
CoM 0265	7.20	11.37	11.54	0.815	12	16	28	0.79

*BLUP* best linear unbiased prediction, *CCSY* commercial cane sugar yield (t/ha), *ASI* AMMI stability index, *rASI* rank of AMMI stability index, *rCY* rank of cane yield, *GSI* genotype selection index, *YREM* yield related environmental maximum.

### AMMI stability analysis for cane and CSS yield

The AMMI ANOVA showed the significance of the mean sum of square due to drought environments, genotypes, and GEI for cane yield, CCS yield, SL, ST, SCW, and non-significance for NMC and sucrose content (Table 5). Main effects of drought environment and genotype and GEI accounted 49.08, 32.59 and 18.33 percent of phenotypic variability for cane yield and 52.45, 28.10 and 19.45 percent of phenotypic variability for CCS yield respectively. With regards to yield component traits, drought environment, genotype and GEI accounted 69.91, 17.13 and 12.96 percent for SL, 63.51, 23.72 and 12.78 percent for SCW and 3.35, 66.39 and 30.26 percent of phenotypic variability for ST suggesting significant manipulative role of genotypes and tillering drought for trait expression.

**Table 5:** Analysis of variance for cane yield, sucrose and component traits by using AMMI model under drought stress in sugarcane.

Parameters	Degree of freedom	CY		CCSY		Sucrose		NMC		SL		SCW		ST	
		MSS	POV	MSS	POV	MSS	POV	MSS	POV	MSS	POV	MSS	POV	MSS	POV
Drought Environment	3	32,072.01**	49.08	636.08**	52.45	51.25**	56.80	5278.84**	45.51	90,383.56**	69.91	7.67**	63.51	0.14**	3.35
Genotypes	15	4260.14**	32.59	68.16**	28.10	3.65**	20.23	606.16**	26.13	4428.58**	17.13	0.57**	23.72	0.54**	66.39
GEI	45	798.60**	18.33	15.73**	19.45	1.38	22.98	219.24	28.35	1116.78**	12.96	0.10**	12.78	0.08**	30.26
IPCA1	17	447.90*	52.09	9.13*	55.33	0.77	51.85	132.67	62.36	747.61	62.40	0.07	59.40	0.05	49.61
IPCA2	15	273.71	28.08	5.69	30.46	0.67	39.60	68.05	28.22	408.61	30.09	0.03	25.73	0.03	31.71
IPCA3	13	222.97	19.83	3.06	14.21	0.17	8.55	20.61	7.41	117.61	7.51	0.02	14.87	0.02	18.68
Residuals	320	242.17		5.30		1.14		179.64		654.26		0.57		0.05	

**significant at 1% level; *significant at 5% level.

*MSS* mean sum of squares, *POV* percent of variation explained by components, *CY* cane yield (t/ha), *GEI* genotype × environment interaction, *IPCA* Interactive principal component axis, *CCSY* commercial cane sugar yield (t/ha), Brix12: juice brix at 12th month, Pol10: sucrose percent at 12th month, *SL* stalk length (cm), *ST* stalk thickness (cm), *SCW* single cane weight (kg), *NMC* number of millable canes.

The variance due to GEI was further partitioned into variance due to signal and noises (Table 6). The portion of variation due to signal occurs with known factors such as genotypes, tillering phase drought, while noise variation was attributed to error associated with model’s unknown factors^22^. In our studies, cane yield and CCS yield recorded 69.68 and 66.31 percent of GEI due to signal and remaining due to noises. This suggests the adequacy of applying the AMMI stability model to understand the GEI.

**Table 6:** Estimates of sum of square due to signals and noises by using AMMI model for drought stress in sugarcane.

Traits	Sum of square		Percent variation	
	GEI_Signal_	GEI_Noise_	GEI_Signal_	GEI_Noise_
CY	25,039.36	10,897.52	69.68	30.32
CCSY	469.35	238.50	66.31	33.69
SL	20,813.59	29,441.51	41.42	58.58
SCW	2.11	2.52	45.50	54.50
ST	1.48	2.21	40.12	59.88

*GEI* genotype × environment interaction, *CY* cane yield (t/ha), *CCSY* commercial cane sugar yield (t/ha), *SL* stalk length (cm), *ST* stalk thickness (cm), *SCW* single cane weight (kg), *NMC* number of millable canes.

The multiplicative component of AMMI models consists of singular value/multiplication factor of IPCA, eigenvector of genotype and environments^49^. The IPCA1 was highly significant for cane yield, CCS yield and non-significant for yield components such as SCW, SL and ST. The IPCA1 for cane yield and CCS yield accounted 52.09 and 55.33 percent of the GEI indicating significant contribution of drought at tillering phase on the performance of genotype and trait expression.

### GGE biplots to visualize GEI for cane and CCS yield

**Figure 2:**
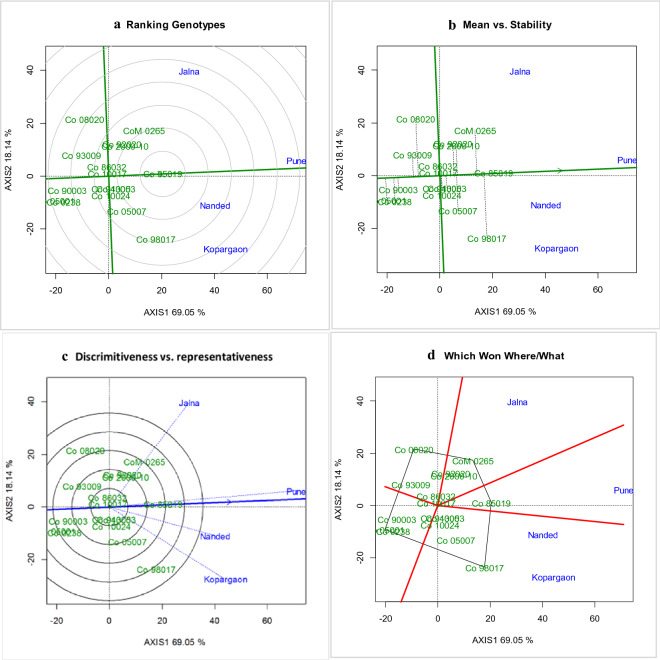
GGE Biplots of for cane yield (t/ha) under tillering phase in drought stress. The data is not transformed, not scaled, environments were centered (Centering = 2) and the biplots based on singular value partition by column metric preserving. The Biplots explained 87.19 percent of G + GE. (**a**) Ranking of genotype GGE biplot depicting the Co 85019 located in first concentric circle as ideal genotype for cane yield (t/ha), (**b**) mean vs stability GGE biplot depicting the Co 85019 located in the direction of the average environmental coordinates as best performing genotype for cane yield (t/ha), (**c**) discriminativeness vs representativeness GGE biplot depicting the longest environment vector and nearer to average environmental coordinates and identified Pune as highly discriminative environment, and (**d**) Which won where/what GGE biplot to visualise the mega environment and environment specific genotypes.

The GGE Biplots were used to visualize the effects of drought, genotypes, and pattern of GEI. The graphical visualization of GGE on Biplots helps to visualize the mega environments, ranking of genotypes, and identification of stable environments^27^. The discriminativeness *vs* representative views of GGE Biplots helps to identify the ideal environments with the highest discriminative power to discriminate the genotypes. The average environmental coordinates (AEC) and test environments are capable of visualizing the type-1 environments, type-2 environments and type-3 environments. The type-1 environments are represented by short vectors with average discriminative power indicating the average performance of genotypes. The type-2 environments are shown as the longest vectors with the highest discriminative powers, capable to discriminate the performance of genotypes. Type-3 environments are represented as the longest vector with large angles, suitable to ill effects of environments. The ideal environments are those having the longest genotypic vector and located on or acute angles to the AEC^28^. In our studies for cane yield and CCS yield, ‘Pune’ has the longest environmental vector with narrow angles to AEC having the highest discriminative power and is considered as the ideal environment for cane yield (Fig. 2c, 3c). The shortest environmental vector observed at ‘Nanded’ indicating the average or similar performance of the genotypes.

**Figure 3:**
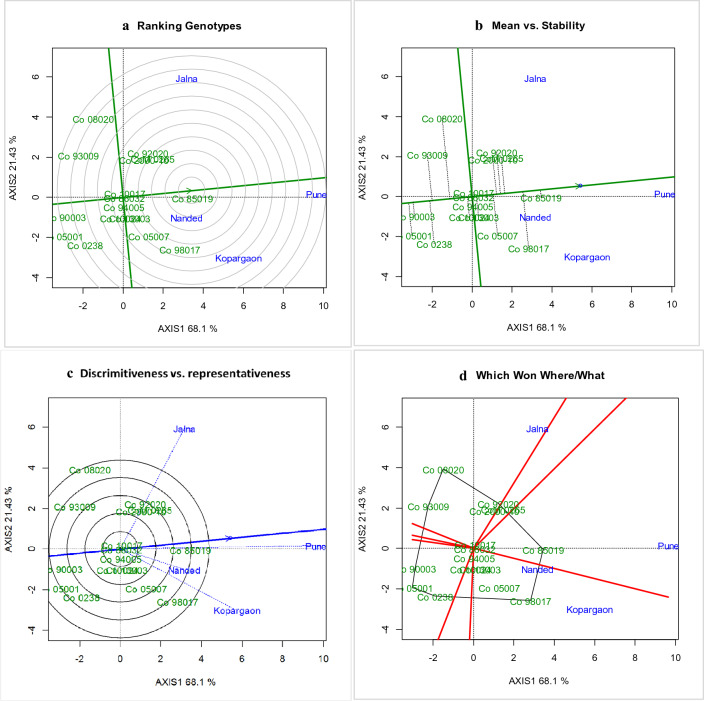
GGE Biplots for CCS yield (t/ha) under tillering phase in drought stress. The data is not transformed, not scaled, environments were centered (Centering = 2) and the biplots based on singular value partition by column metric preserving. The Biplots explained 89.53 percent of G + GE. (**a**) Ranking of genotype GGE biplot depicting the Co 85019 located in first concentric circle as ideal genotype for CCS yield (t/ha), (**b**) Mean vs stability GGE biplot depicting the Co 85019 located in the direction of the average environmental coordinates as best performing genotype for CCS yield (t/ha), (**c**) discriminativeness vs representativeness GGE biplot depicting the longest environment vector and nearer to average environmental coordinates and Pune was identified as highly discriminative environment specific genotypes.

The mean *vs* stability biplots helps to understand the mean performance of genotypes across drought environments. The genotypes located or nearest and in the direction of AEC are considered as ideal and best performing genotypes^28^. The best performing genotype for cane yield (Fig. 2b), was Co 85019 as the most stable and better performing clone followed by Co 98017 and CoM 0265 (Co 85019 > Co 98017 > CoM 0265) and poor performing genotypes were Co 05001, Co 0238 and Co 90003 (Co 0238 < Co 05001 < Co 90003). Similarly for CCS yield (Fig. 3b), Co 85019 was identified as better performing stable clone followed by Co 98017 and CoM 0265 (Co 85019 > Co 98017 > CoM 0265). The poor performing clones were Co 05001, Co 90003 and Co 0238 (Co 0238 < Co 90003 < Co 05001). From our studies, it showed that Co 85019 located on or near AEC and considered as the most stable high performing genotype whereas CoM 0265 and Co 98017 recorded high yield but were located away from the AEC, hence considered as less stable varieties.

The ranking of genotype on Biplots helps to visualize the ideal genotypes based on their locations in the concentric circle^28^. The entry Co 85019 occupied the first concentric circle (Fig. 2a, 3a) for cane yield and CCS yield indicating its stable performance across environments, while Co 98017, Co 05007, Co 2000-10, Co 92020, Co 10024, Co 10017, Co 86032, CoM 0265 located on the third concentric circle for cane yield are considered as less stable varieties under drought stress.

‘Which Own Where Biplots’ helps to visualize the mega environments and identification of superior genotypes to the specific drought environment. The genotypic mean plotted against first interaction principal components where the genotype is represented by a line and IPCA as slope, these biplots are known as ‘which own where biplots’^28^. The irregular polygons divide the biplots into the vector and help in the identification of the best performing genotype for each environmental sector. In our studies, Co 85019 was the best performing genotype in Pune, CoM 0265 was the best at Jalna and Co 98017 was the best genotype at Kopargaon and Nanded centres for cane yield and CCS yield (Fig. 2, 3d).

### AMMI and YREM based stability indices for cultivar performance to cane yield and CCS yield in sugarcane

The lowest AMMI stability value (ASV) estimated from the IPCA axis and IPCA scores indicate the most stable variety^18^. The lowest ASV for cane yield was recorded by Co 05001, Co 0238, Co 13003 and recorded the lowest cane yield of 54.11, 57.93 and 84.06 t/ha respectively under acute tillering phase drought stress. The high cane yielding varieties such as Co 85019, Co 98017 and CoM 0265 recorded the ASV value of 0.4395, 1.8370 and 0.9810 respectively. For CCS yield, the lowest ASV was recorded by Co 05001, Co 86032 and Co 90003. The high CCS yielding clones Co 85019, Co 98017 and Co 05007 recorded the ASV of 0.715, 1.539 and 1.051 respectively. The high genotypic variability and high coefficient variability are normally observed under stressed experiments^35^. The performance of high yielding genotypes depends on genotype recovery status during the grand growth stage after the acute formative phase drought stress^13^.

The genotype selection indices (GSI) were estimated based on the sum of ranks of ASV and ranks of genotypes derived from cane yield and CCS yield. The lowest value of GSI indicates the most stable and high performing genotypes across environments^21,51^. For cane yield, the lowest GSI was recorded by Co 85019, Co 13003 and Co 94005, suggesting that these genotypes are combined with high stability and high cane yield across environments. For CCS yield Co 85019, Co 86032 and Co 13003 recorded the lowest GSI suggesting that these genotypes are combined with high stability and high CCS yield across environments. Based on GSI and trait mean for cane yield and CCS yield, Co 85019 recorded the highest cane and CCS yield of 104.26 t/ha and 13.85 t/ha with lowest GSI. Hence, Co 85019 is considered as the most stable high yielding variety under tillering phase drought stress.

The yield relative to the environmental maximum (YREM) is a simple, intuitive, cultivar independent factor which helps the breeders to select the cultivars in presence of unpredictable crossover G × E interactions. In the absence of G × E interaction crossover, the value of YREM is 1.0 indicating the most stable variety. The YREM is 0.9 implies 10 percent of yield loss due to the unpredictable G × E crossover interaction^50^. The clone Co 85019 recorded the highest YREM of 0.87 and 0.91 for cane yield and CCS yield respectively. Other clones such as Co 98017 recorded the superior YREM of 0.86 and 0.89 indicating that, 14 and 11 percent loss for cane yield and CCS yield due to G × E crossover interaction.

## Discussion

Sugarcane is a major agro industry-based cash crop and a major source of income for millions of farmers in the world. The global climatic changes and aberrations in weather parameters causes significant variation in quantum and frequency of rainfall in major sugarcane growing states^1–3,55–57^. The meteorological data analysis has shown the recurrence of drought with high frequency in many parts of the world^4,5,58^. The rainfall distribution across the world shows that maximum precipitation was received during monsoon seasons coinciding with June to September in the northern hemisphere and December to May in the southern hemisphere^5–8^. Hence, sugarcane crop inevitably gets exposed to drought stress due to its trans-seasonal long duration nature major sugarcane growing countries. Earlier research carried out at ICAR Sugarcane Breeding Institute identified several drought stress tolerant lines^15,33,59^ and we selected fourteen drought tolerant clones for multi-location evaluation in the prevailing drought stress affected regions to identify the drought tolerant clones suitable for cultivation in Peninsular India.

Tillering phase of sugarcane crop is the most sensitive phase to moisture stress causing significant reduction in cane yield through the reduction in its component traits^9,11–13,30^. Hence, the fourteen drought tolerant clones and two standard varieties were evaluated by imposing drought stress through withholding the irrigation from 90 to 150 days. The performance of the clones was compared with the widely grown wonder variety Co 86032 which covered over 50% area and the high biomass yielding local variety CoM 0265, notified for cultivation in Maharashtra state and cultivating in 18 percent of area^34^. The Co 85019 recorded the cane yield of 104.26 t/ha across locations with the improvement of 26.21 and 5.61 percent improvement over the two standards respectively. The clone Co 98017 recorded 101.06 t/ha with an improvement of 22.34% and 2.37% over Co 86032 and CoM 0265 respectively. The superior performance of Co 85019 and Co 98017 is in confirmation with the previous report for cane yield and sucrose content under drought stress in Coimbatore^15,33^. Co 85019 was identified as salinity tolerant clone based on physiological and cane yield/biomass accumulation under salinity stress^37–39^ and further used for characterization of genes^40^. Among the thirty-three clones evaluated under water limited condition, Co 85019 was identified for high water use efficiency, water productivity^35^ and, high physiological efficiency for related traits such as canopy temperature deficient, chlorophyll efficiency, leaf rolling index^36^ .

BLUP is the standard method of estimating the random effects followed in both animal and plant breeding. The comparison of cultivar performance by both AMMI and BLUP in faba bean showed that BLUP outperformed in the prediction of superior genotypes than AMMI^52^. The prediction of yield estimate by BLUP was found superior as compared to least square and BLUE methods in soybean^60^. Similar results were obtained for genetic values in maize multi-environmental traits^61^. In our studies, the estimate of multi-environment BLUP showed that Co 85019 recorded the highest BLUP mean for cane yield (99.41 t/ha) and CCS Yield (12.93 t/ha) followed by Co 98017 (99.31t/ha of cane yield and 12.92 t/ha of CCS yield) as compared to better standard CoM 0265, which recorded 93.37 t/ha of cane yield and 11.37 t/ha of CCS yield under tillering phase drought stress. The cane and CCS yield showed a significant positive genetic correlation with NMC, SL, ST, SCW and this is in confirmation with previous reports^62^.

AMMI stability model is widely used to understand the pattern of GEI and to identify the stable cultivars from target environments^22^. The AMMI ANOVA showed remarkable contributions from drought environments, genotypes, and GEI for cane yield, CCS yield and their components. Further, the mean sum of squares due to GEI were significant for cane yield and CCS yield, SL, ST and SCW, indicating the significance of tillering phase drought stress on genotypic expressions. The non-significant mean sum of squares was recorded for sucrose and NMC indicating that expression of these traits was not influenced by tillering phase drought stress. This is in confirmation with previous reports for cane yield^63^ and CCS yield^64–66^. The sum of square due to GEI_Signal_ is larger than the GEI_Noise_ for cane and CCS yield, SL, ST and SCW due to larger contribution from additive main effect of genotype and tillering phase drought stress environments and reiterated the use of AMMI model for understanding the pattern of GEI.

The biplots of GGE helps to elucidate the interrelation among environments, ranking of genotypes and to identify the better performing genotype in a given environment^27^. From our studies, discriminativeness *vs* representative showed that ‘Pune’ as the ideal environment by virtue of its weather parameters. The relatively cooler climate in ‘Pune’ during the drought (daily average mean temperature of 29.45 °C and 29.41 °C during 2018 and 2019) and the drought recovery phase (daily average mean temperature of 25.37 °C and 26.01 °C) attributed appropriate environmental effect in crop expression. Further IPCA1 vs IPCA2 of AMMI biplots also displayed diverse nature of the four environments. The ranking of genotypes showed that Co 85019 was the most stable variety and Co 98017 was a high yielding variety with relatively lesser stability over Co 85019. The IPCA1 vs cane yield AMMI Biplot showed that Co 85019 and Co 98017 were high yielding with relatively less GEI for tillering phase drought stress and considered as stable varieties.

The parameters ASV, GSI and YREM are commonly used to identify the stable varieties in multi-environment trials^18,19,50,67^. The lower values of ASV indicate the most stable variety^18^ and the lowest GSI could be inferred as high performing stable genotypes^21^. YREM identified the stable genotypes and yield loss due to G × E crossover interactions^50,51^. In this study , Co 85019 was ranked sixth for ASV, ranked first for GSI and recorded the highest YREM of 0.87 for cane yield and 0.91 for CCS yield indicating that 13 percent of cane yield and nine percent of CCS yield were lost due to G × E crossover interaction. Similarly, Co 98017 recorded YREM of 0.86 and 0.89 suggesting loss of 14 and 11 percent of cane yield and CCS yield due to G × E crossover interactions respectively.

## Conclusions

The drought tolerant clones identified over years at ICAR-Sugarcane Breeding Institute, Coimbatore were evaluated under tillering phase drought stress by withholding the irrigation for 90-150 days at four locations experiencing water deficit stress across Maharashtra, India. The weather data clearly showed nil rainfall, low relative humidity and high daily temperature during the drought imposition period. The AMMI biplots with interaction PCA1 and PCA2 and with yield revealed high discriminative environments. The multi-environment BLUP, AMMI, GGE Biplots and YERM analysis clearly showed the stable performance of Co 85019 and superior performance of Co 98017 for cane yield and CCS yield. The test clone Co 05007 also showed superior performance for CCS yield. These three genotypes could be deployed for the drought prone regions in Peninsular India, while could serve as parents in genetic improvement of drought tolerance in view of climate change.

## Supplementary Information

Supplementary Information.

## Abbreviations

CCS: Commercial cane yield
NMC: Number of millable canes
SL: Millable stalk length
ST: Stalk thickness
SCW: Single cane weight
AMMI: Additive main effects and multiplicative interactive effects stability analysis model
GGE: Genotype + genotype × environment interaction
ANOVA: Analysis of variance
GEI: Genotype × environment interaction
IPCA: Interactive principal component axis
ASV: AMMI stability value
GSI: Genotypic selection index
YREM: Yield related environmental Maximum,
BLUP: Best linear unbiased prediction
