# Supplementary material for: Delineation of genotype × environment interaction for identification of stable genotypes for tillering phase drought stress tolerance in sugarcane
Source: Sci Rep. 2021 Sep 20;11:18649. doi: 10.1038/s41598-021-98002-y (PMC8452706; doi:10.1038/s41598-021-98002-y)
Supplement: Supplementary file 1 — Supplementary Information. [file 41598_2021_98002_MOESM1_ESM.docx]

**Supplementary table 1. List of sugarcane drought tolerant clones, their parentage and selection based on the research work carried out at ICAR-Sugarcane Breeding Institute, Coimbatore**

The newly developed breeding lines are evaluated for drought stress every year at ICAR-Sugarcane Breeding Institute, Coimbatore by imposing the drought stress by withdrawing the irrigation from 90 days to 150 days after planting (tillering phase). The results are presented in the Institute Research Council (IRC) meeting and Annual Reports of ICAR-Sugarcane Breeding Institute, Coimbatore. Besides, many peer-reviewed research articles are published from this institute on abiotic stress tolerances. We have selected the abiotic stress tolerance clones by referring to the Annual Report, ICAR-Sugarcane Breeding Institute, Coimbatore, and peer-reviewed research publications.

| **Sl No** | **Sugarcane**  **Varieties** | **Parentage** |  |
| --- | --- | --- | --- |
| 1 | Co 85019 | Co 7201 × Co 775 | 1. Co 85019 was identified as highest water use efficiency and water productivity clone under limited irrigation condition (Tayade et al., 2020) 2. Co 85019 physiological efficiency such as canopy temperature deficient, chlorophyll efficiency, leaf rolling index and cane yield under limited irrigation condition (Arun Kumar et al., 2020) 3. Highest biomass accumulation under salinity stress (Vasantha et al., 2020) 4. Co 85019 was identified as salinity stress tolerant based on physiological parameters such as chlorophyll fluorescence, relative water content, cane parameters such as stalk length, stalk weight, number of millable canes and cane yield in replicated block trial at Coimbatore (Brindha et al., 2019) 5. Co 85019 recorded the highest biomass accumulation under salinity and drought stress at Coimbatore (Vasantha et al., 2017) 6. Co 85019 was used for characterization of genes conferring to the salinity stress tolerance (Brindha et al., 2021) 7. Co 85019 was identified as drought tolerant based cane yield under formative phase drought stress (Hemaprabha et al., 2013) |
| 2 | Co 0238 | CoLk 8102 × Co 775 | Co 0238, a wonder variety occupying more than 80 percent of area under subtropical regions (Ram and Hemaprabha, 2020). |
| 3 | Co 05001 | Co 94008 General cross | Co 05001 was identified as drought tolerant clone (Gomathi, Annual Report, 2010-11, ICAR-Sugarcane Breeding Institute, Coimbatore) |
| 4 | Co 05007 | Co 94008 | Co 05007 was identified as drought tolerant clone (Gomathi, Annual Report, 2010-11, ICAR-Sugarcane Breeding Institute, Coimbatore) |
| 5 | Co 08020 | Co 775 × Co 1148 | Co 08020 was identified as drought tolerant clone (Gomathi, Annual Report, 2013-14, ICAR-Sugarcane Breeding Institute, Coimbatore) |
| 6 | Co 10017 | CoC 671 × IG91-1100 | Co 10017 was evaluated under drought stress and satisfactory crop stand (Gomathi, Annual Report, 2016-17, ICAR-Sugarcane Breeding Institute, Coimbatore) |
| 7 | Co 10024 | Co 92024 General cross | Co 10024 was evaluated under drought stress and satisfactory crop stand (Gomathi, Annual Report, 2016-17, ICAR-Sugarcane Breeding Institute, Coimbatore) |
| 8 | Co 13003 | Co 86011 × CoT 8201 | Co 13003 was identified as drought tolerant clone (Gomathi, Arunkumar, Krishnapriya, Annual Report, 2018-19, ICAR-Sugarcane Breeding Institute, Coimbatore) |
| 9 | Co 2000-10 | Co 88017 × CoC 773 | 1. Co 2000-10 was identified as drought tolerant based cane yield under formative phase drought stress (Hemaprabha et al., 2013) 2. Co 2000-10 was identified as drought tolerant based cane yield under formative phase drought stress (Hemaprabha et al., 2008) |
| 10 | Co 90003 | L-78-767 × E79-269 | Co 90003 was identified as drought tolerant based cane yield under formative phase drought stress (Hemaprabha et al., 2013) |
| 11 | Co 92020 | Co 7201 × Co 1307 | Co 92020 was identified as drought tolerant clone. (Gururaja Rao Annual Report, 1996-97, ICAR-Sugarcane Breeding Institute, Coimbatore) |
| 12 | Co 93009 | Co 678 × Co 775 | Co 93009 was identified as salinity tolerant clones. (Sanjiv Reddy, Vasantha, Gomathi, Annual Report, 1999-2000, ICAR-Sugarcane Breeding Institute, Coimbatore) |
| 13 | Co 94005 | Co 7201 × Co 775 | Co 94005 was identified as salinity tolerant clone. (Vasanth and Ramanujam, Annual Report, 1998-99, ICAR-Sugarcane Breeding Institute, Coimbatore) |
| 14 | Co 98017 | Co 8316 × Co 8213 | Co 98017 was identified as drought tolerant based cane yield under formative phase drought stress (Hemaprabha et al., 2008) |
|  | **Standards** |  |  |
| 1 | Co 86032 | CoC 671 × Co 62198 | Notified variety for cultivation in Peninsular India. |
| 2 | CoM 0265 | Co 87044 GC | Notified variety for cultivation in Maharashtra state, India. |

**Supplementary table 2. Experimental locations, sugar factory details and crop years of the ten drought environments**

| **Locations** | **Sugar factory** | **Year** | **Crop Year** |
| --- | --- | --- | --- |
| Kopargaon | M/s Sahakari Maharishi Shankararao Kolhe Sahakari Sakkara Sangha, Sanjivaninagar, Kopargaon, Ahmednagar District | 2018 | Plant Crop |
|  |  | 2019 | Ratoon |
| Jalna | M/s Karmayogi Ankushrao Tope Samarth Sahakari Sakkara Kharkana, Jalna | 2018 | Plant Crop |
|  |  | 2019 | Plant Crop |
|  |  | 2019 | Ratoon |
| Nanded | M/s Bhaurao Chavan Sahakari Sakkara Kharkana, Nanded | 2018 | Plant Crop |
|  |  | 2019 | Plant Crop |
| Pune | Vasantdada Sugar Institute, Pune | 2018 | Plant Crop |
|  |  | 2019 | Plant Crop |
|  |  | 2019 | Ratoon |

**Supplementary table 3. Chemical properties of soil**

| **Sl**  **No** | **Parameters** | **Kopargaon** | **Jalna** | **Nanded** | **Pune** |
| --- | --- | --- | --- | --- | --- |
| 1 | Soil pH | 7.95 | 8.23 | 7.75 | 8.12 |
| 2 | Soil Electrical conductivity (dS/cm) | 0.67 | 0.50 | 0.19 | 0.41 |
| 3 | Organic carbon | 0.39 | 0.38 | 0.35 | 0.63 |
| 4 | Nitrogen (kg/ha) | `254.02 | 238.33 | 245.00 | 134.85 |
| 5 | Phosphorous (kg/ha) | 10.15 | 11.67 | 13.42 | 2.86 |
| 6 | Potassium (kg/ha) | 1087.26 | 1171.84 | 479.64 | 583.76 |

Soil test analysis was carried out at Vasantdada Sugar Institute, Pune

**Supplementary table 4. Weather data parameters recorded during drought imposition period at tillering, drought recovery and maturity stages in ten test drought environments**.

| **Crop Stage** | **Locations** | **Total Rainfall (mm)** | | **Daily mean relative humidity (%)** | | **Daily mean**  **temperature** | |
| --- | --- | --- | --- | --- | --- | --- | --- |
|  |  | **2018** | **2019** | **2018** | **2019** | **2018** | **2019** |
| Drought imposition  (Tillering phase)  (April 01 to May 30) | Kopargaon | 1.21 | 6.26 | 30.34 | 29.82 | 31.06 | 30.76 |
|  | Jalna | 4.18 | 3.69 | 21.89 | 21.27 | 33.42 | 33.13 |
|  | Pune | 4.79 | 2.45 | 39.93 | 38.44 | 29.45 | 29.41 |
|  | Nanded | 23.48 | 3.95 | 21.65 | 18.69 | 34.91 | 35.07 |
| Grand growth period  (Recovery phase)  (June 01 to August 31) | Kopargaon | 652.01 | 884.77 | 78.88 | 77.74 | 25.37 | 26.01 |
|  | Jalna | 483.90 | 397.07 | 70.93 | 67.65 | 26.89 | 28.02 |
|  | Pune | 652.01 | 884.77 | 78.88 | 77.74 | 25.37 | 26.01 |
|  | Nanded | 599.06 | 421.79 | 73.01 | 66.03 | 27.12 | 28.94 |
| Maturity period  (September 01 to December 31) | Kopargaon | 4.74 | 36.32 | 34.96 | 78.28 | 22.11 | 20.65 |
|  | Jalna | 9.67 | 26.32 | 35.21 | 76.50 | 22.41 | 20.76 |
|  | Pune | 11.20 | 46.90 | 51.51 | 80.81 | 21.08 | 21.07 |
|  | Nanded | 7.09 | 10.46 | 38.88 | 69.53 | 23.21 | 21.97 |

**Supplementary table 5. List of characters recorded from the drought experiments in Maharashtra**

| **Sl No** | **List of observations recorded** |
| --- | --- |
| 1. | Germination (%) |
| 2. | Number of tillers at 90 days: Total of number of tillers were counted for each plot and number of tillers (000/ha) was estimated |
| 3. | Leaf rolling scores at 150 days: Scored in 1-9 scales as described d by system of standard evaluation system for rice, International Rice Research Institute, Los banos, Phillippines (2002). The leaves above visible dewlab showing leaf rolling on 150^th^ day were scored. |
| 4. | Leaf tip drying score at 150 days: Scored in 1-9 scales as described d by system of standard evaluation system for rice, International Rice Research Institute, Los Banos, Phillippines (2002). The leaves above visible dewlab showing leaf tip drying on 150^th^ day were scored. |
| 5. | Stalk length at 300 and 360 days after planting: Measured from ground level to node of the first visible dewlab from top of the canopy. The observation were averaged over five canes and used for data analysis |
| 6. | Stalk diameter at 300 and 360 days after planting: Measured at middle of the millable cane and averaged over five canes |
| 7. | Stalk weight at 300 and 360 days after planting, averaged over five canes |
| 8. | Juice brix, Pol, CCS (%) and Juice purity at 300 days: Five canes were sampled randomly from a clump, shredded using sugarcane crusher and juice was extracted. The juice was used measuring the brix, pol and, estimation of CCS (%) and juice purity. |
| 9. | Number of millable canes (NMC) at 360 days: Number of millable canes was counted in middle four rows and NMC (000/ha) was estimated. |
| 10. | Cane yield (t/ha): Plot yield was determined by harvesting the canes from the middle four rows and cane yield (t/ha) was estimated |
| 11. | Commercial cane sugar yield (CCS yield; t/ha) at 360 days: CCS yield (t/ha) was estimated in from cane yield and CCS (%). |
